# Engaging with Indigenous Australian communities for a human papilloma virus and oropharyngeal cancer project; use of the CONSIDER statement

**DOI:** 10.1186/s12874-020-00981-5

**Published:** 2020-04-25

**Authors:** Joanne Hedges, Gail Garvey, Zell Dodd, Warren Miller, Terry Dunbar, Cathy Leane, Amanda Mitchell, Isaac Hill, Lisa Jamieson

**Affiliations:** 1 The University of Adelaide, Adelaide, Australia; 2 Menzies School of Health Research, Darwin, Australia; 3 Ceduna Kooniba Aboriginal Health Service Aboriginal Corporation, Ceduna, Australia; 4 Australian National University, Sydney, Australia; 5 South Australian Health, Adelaide, Australia; 6 Aboriginal Health Corporation South Australia, Adelaide, Australia; 7 Aboriginal Health Council South Australia, Adelaide, Australia

**Keywords:** Research, Indigenous, Consultation, Engagement, Recruitment, Focus groups, Aboriginal community controlled health Organisations, CONSIDER statement

## Abstract

**Background:**

The prevalence of oral HPV infection and HPV-related oropharyngeal squamous cell carcinoma (OPSCC) among Indigenous Australians is unknown. This paper outlines the engagement, consultation and recruitment strategies for a study involving **investigation of** HPV and OPSCC among Indigenous South Australians, based on the consolidated criteria for strengthening the reporting of health research involving Indigenous Peoples (CONSIDER) statement.

**Methods:**

Initial consultations with all interested Aboriginal Community Controlled Health Organisations (ACCHOs) were done throughout 2014 and 2015. This resulted in a funding application submitted that reflected Indigenous community views and inputs in study design and methodology, and which included nine Indigenous investigators. Once funding was received, community consultation was again undertaken, with six ACCHOs providing structures, strategies and recommendations for how recruitment **for participants taking part in the study** should be undertaken. Staff were hired (*n* = 6), with non-Indigenous staff (*n* = 3) undertaking extensive cultural competency training. An Indigenous Reference Group was established to provide oversight and cultural guidance. Recruitment **of Indigenous participants by trained field officers** occurred between Feb 2018 and Dec 2018, with *n* = 1011 recruited. Qualitative records summarising research staff contact with ACCHOs and participants were documented. These records, together with field trip notes, key ACCHO stakeholder reflections and research staff comments, were reviewed to summarise the culturally sensitive strategies that appeared to work most successfully to facilitate ACCHO and participant buy-in.

**Results:**

Findings were documented against the CONSIDER statement’s research reporting framework of governance: relationships, prioritization, methodologies, participation, capacity, analysis and findings, and dissemination. The apparent success of the community engagement processes were then conceptualised into five domains: (1) engaging with ACCHOs as equal partners very early in the research process; (2) having an Indigenous Reference Group; (3) ACCHOs actively promoting the study; (4) having a flexible agenda responsive to broader environment demands and; (5) including Indigenous capacity building.

**Conclusions:**

Consultation and engagement with all sectors of the Indigenous community are essential in any research, **especially a project involving HPV and OPSCC**. Enabling local Indigenous staff to provide cultural guidance throughout the research process is helpful. Research that is culturally respectful and in partnership with Indigenous groups can be embraced when the research is collaborative and has clear translational benefits. The CONSIDER statement is a useful checklist against which to assess Indigenous health research processes. **In future, the findings may be useful to yield important Aboriginal population estimates for both oral HPV infection and OPSCC. This may serve to convince funding bodies to provide health promotion personnel in the field of oral health, specifically OPSCC, in ACCHOs.**

## Background

Research involving Aboriginal and Torres Strait Australians (hereafter respectfully termed ‘Indigenous’) should be shaped by a number of guiding principles, such as those recommended by the National Health and Medical Research Council (NHMRC) [1], the Australian Institute of Aboriginal and Torres Strait Islander Studies [2] and the Wardliparingga Aboriginal Research Unit based in the South Australian Health and Medical Research Institute [3]. These principles stipulate that Indigenous communities need to be engaged in all aspects of research undertaken in their communities and organisations. It is also important to ensure that any research processes embrace the shared values of Indigenous peoples and their communities, including their diversity, priorities, needs and aspirations. Central to this is that the research is of benefit to Indigenous populations with, ideally, the original idea for the research coming from Indigenous groups in the first instance. It is recommended that meaningful engagement and reciprocity between researchers and Indigenous groups occurs early and is sustained throughout the research process. All Indigenous participants and stakeholder groups need to be regarded as equal partners in the research engagement process.

Huria and colleagues recently developed the Consolidated Criteria for Strengthening Reporting of Health Research involving Indigenous Peoples (CONSIDER) statement [4]. This involved reviewing seven national and international statements and guidelines about Indigenous health research from Aboriginal and Torres Strait Islanders in Australia, First Nation and Metis peoples in Canada, Native Hawaiians in Hawaii, Māori in New Zealand, Taiwan Indigenous Tribes in Taiwan, First Nations peoples in the United States and Sami peoples in Northern Scandinavia. The authors then conducted a meta-synthesis to construct a comprehensive checklist for reporting of research involving Indigenous persons. This checklist comprises eight domains which, in turn, comprise 17 criteria. The domains are: (1) governance, (2) relationships, (3) prioritization, (4) methodologies, (5) participation, (6) capacity, (7) analysis and findings, and (8) dissemination. Utilising an approach such as this is especially important when researching sensitive topics such as highly infectious viruses such as human papillomavirus and resulting conditions, including oropharyngeal squamous cell carcinomas.

Human papillomaviruses (HPVs) are double-stranded DNA viruses that grow in the stratified epithelia of skin and mucous membranes. Prior to implementation of a vaccination to protect against HPV, a restricted number of high risk HPV genotypes were the most common sexually transmitted infections in Australia [5]. These HPVs are a precursor to a range of cancers in both females and males, particularly cervical cancer, other anogenital cancers and oropharyngeal cancer [6]. In Australia, the incidence of HPV-related oropharyngeal squamous cell carcinoma (OPSCC) increased among men and women by an estimated 1% per year from 1982 to 2005 [7]. Indigenous Australians are over-represented in almost all head and neck cancers [8]. However, the prevalence of oral HPV infection, and HPV-related OPSCC, among Indigenous Australians is unknown.

This paper outlines the engagement, consultation and recruitment strategies used in a study investigating the association of oral HPV infection and OPSCC among Indigenous Australians residing in South Australia. Findings are documented against the CONSIDER statement’s research reporting framework.

## Methods

**Fig. 1:**
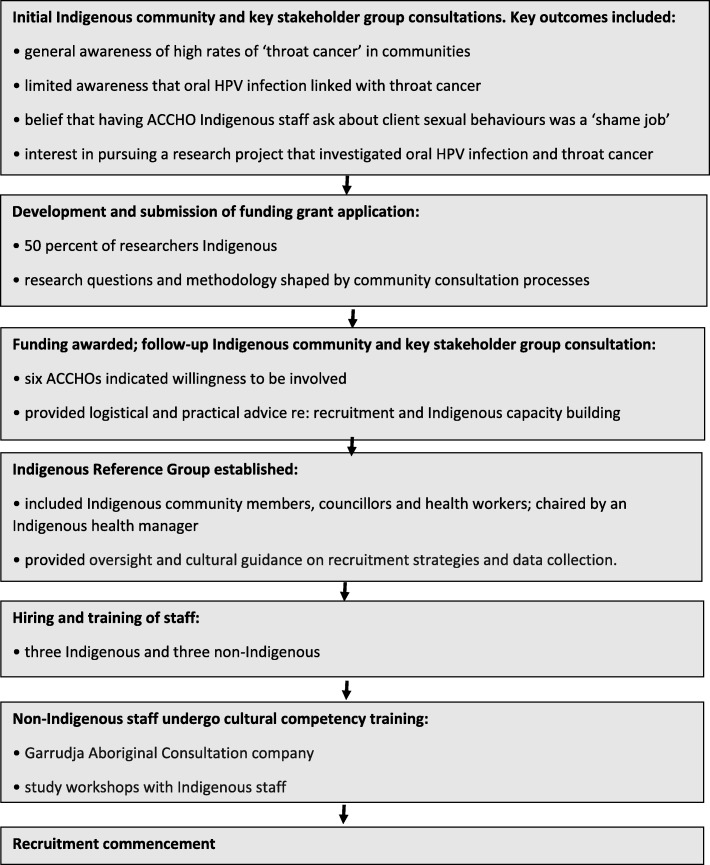
Schema outlining the community consultation, grant development and other study-related processes leading to recruitment

A number of processes, informed by the NHMRC guidelines, were implemented to ensure the study, from conception to recruitment, embraced principles that helped facilitate the building of reciprocal and respectful relationships between the Indigenous stakeholders and the researchers. These are outlined below and illustrated in a schema (Fig. 1). They are also documented against the CONSIDER framework (Table 1).

**Table 1:** Consolidated Criteria for Strengthening Reporting of Health Research involving Indigenous Peoples (CONSIDER) checklist

Domain	Criteria	Indigenous Oral HPV-Oropharyngeal Cancer study
Governance	a. Describe partnership agreements between the research institution and Indigenous-governing organization for the research.	● Formal letters of support from participating Aboriginal Community Controlled Health Organisations (ACCHOs)
	b. Describe accountability and review mechanisms within the partnership agreement that addresses harm minimization	● Harm minimisation included as part of informed consent processes and the human research ethics requirements
	c. Specify how the research partnership agreement includes protection of Indigenous intellectual property and knowledge arising from the research, including financial and intellectual benefits generated	● Protection of Indigenous intellectual property and knowledge emphasized in each of the community consultation and engagement sessions, and also individually with participants through the informed consent process
Prioritisation	a. Explain how the research aims emerged from priorities identified by either Indigenous stakeholders, governing bodies, funders, non-government organization(s), stakeholders, consumers, and empirical evidence	● Idea for the study first portrayed by Indigenous community members following feedback session for another health research project by the study investigators ● Extensive community engagement and consultation to refine the study aims
Relationships (Indigenous stakeholders/participants/research team)	a. Specify measures that adhere and honour Indigenous ethical guidelines, processes, and approvals for all relevant Indigenous stakeholders, recognizing that multiple Indigenous partners may be involved	● Ethics approval sought and obtained from two separate human research ethics committees; University of Adelaide and Aboriginal Health Council of South Australia
	b. Report how Indigenous stakeholders were involved in the research processes (i.e., research design, funding, implementation, analysis, dissemination/ recruitment).	● Initial consultations with all interested ACCHOs. ● Funding application submitted that reflected Indigenous community views and inputs in study design and methodology ● Nine Indigenous investigators included. ● Community consultation repeated once funding was received, with ACCHOs providing structures, strategies and recommendations for recruitment and data collection. ● Staff hired (3 Indigenous, 3 non-Indigenous), with non-Indigenous staff undertaking cultural competency training. ● An Indigenous Reference Group established to provide oversight and cultural guidance.
	c. Describe the expertise of the research team in Indigenous health and research	● Nine Indigenous investigators all recognised leaders in their respective Indigenous health research fields ● Three of the nine non-Indigenous research team extensive experience working with Indigenous communities
Methodology	a. Describe the methodological approach of the research including a rationale of methods used and implication for Indigenous stakeholders e.g., privacy and confidentiality (individual and collective)	● Large scale observational study with follow-up after 12 and 24 months ● All data de-identified, with field staff not analysing data once entered in database
	b. Describe how the research methodology incorporated consideration of the physical, social, economic and cultural environment of the participants and prospective participants. (e.g., impacts of colonization, racism, and social justice). As well as Indigenous worldviews	● Baseline questionnaire (as consequence of community consultation) included items pertaining to experiences of racism, major life events (incarceration, death, child removal), social disadvantage and access to health services
Participation	a. Specify how individual and collective consent was sought to conduct future analysis on collected samples and data (e.g., additional secondary analyses; third-parties accessing samples (genetic, tissue, blood) for further analyses).	● Consent forms explicitly stating that no third parties will have access to samples or data ● Any secondary analyses/long-term follow-up of study participants will only be conducted by research team
	b. Described how the resource demands (current and future) placed on Indigenous participants and communities involved in the research were identified and agreed upon including any resourcing for participation, knowledge, and expertise	● Participating ACCHOs provided support only when their resources allowed ● Participants were made aware, during the informed consent process, of the time commitments to being involved in the study
	c. Specify how biological tissue and other samples including data were stored, explaining the processes of removal from traditional lands, if done, and of disposal.	● Saliva samples genotyped for HPV once and then destroyed ● Data stored on password-protected computer software at the University of Adelaide for 15 years
Capacity	a. Explain how the research supported the development and maintenance of Indigenous research capacity	● Study employed three Indigenous staff (one the project manager) ● Indigenous research assistants, including those volunteered by ACCHOs, trained in research skills, ethics principles, data collection, data checking, data filing and disseminating research findings back to community. ● ACCHO staff given opportunity to represent the study at national and international meetings.
	b. Discuss how the research team undertook professional development opportunities to develop the capacity to partner with Indigenous stakeholders	● ACCHOs and study participants were able to bolster their knowledge of HPV infection and OPSCC, and the links between the two, through free and frank conversations with the research team. ● Knowledge-sharing was two-way, with substantial benefits for the non-Indigenous research staff in being included in Indigenous consultative processes and learning from ACCHOs and study participants.
Analysis and interpretation	a. Specify how the research analysis and reporting supported critical inquiry and a strength-based approach that was inclusive of Indigenous values.	● Research analysis still in its infancy, but thus far has included all key Indigenous stakeholders and Indigenous researchers as co-authors in publications. This has enabled Indigenous values and perspectives to contribute to interpretation of the study findings
Dissemination	a. Describe the dissemination of the research findings to relevant Indigenous governing bodies and peoples.	● Still in its infancy, but thus far has included presentations to key Indigenous stakeholder and other Indigenous community groups, presentations at international conferences (Indigenous project manager and two ACCHO staff)
	b. Discuss the process for knowledge translation and implementation to support Indigenous advancement (e.g., research capacity, policy, investment).	● The findings will hopefully support increased resourcing for Aboriginal Health Workers to be specifically employed to facilitate increased understanding of the links between HPV and OPSCC ● This will, in turn, facilitate increased capacity for other areas of research involving HPV, including translation to policy for screening for HPV-related oral cancers

### Community consultation

Initial consultations with all interested Aboriginal Community Controlled Health Organisations (ACCHOs) in South Australia were conducted throughout 2014 and 2015. The broad topic related to the increasing evidence of associations between HPV and OPSCC, the high prevalence of both in the Indigenous Australian population, and how community understanding of the HPV-OPSCC association was limited. Specific feedback included: (1) most people knew of someone who had either been diagnosed with ‘throat’ cancer (the preferred term for OPSCC among the groups consulted) or who had presented with the signs/symptoms but elected no further treatment; (2) there was generally no knowledge of oral transmission processes of HPV infection prior to consultation, with communities largely feeling it was paramount that this information be portrayed, in simple language, to the general community (particularly younger community members); (3) the high risk of mortality from throat cancer was also not recognised, and was considered to be another important message to convey; particularly given that prevention through vaccination could be so effective; (4) unanimous consensus that screening through main stream, general population health clinics would yield a limited number of Indigenous clients; (5) Aboriginal health practitioners believed it was **their remit to be trained to conduct** the collection of saliva samples (to test for oral HPV infection), with the general view being that it was an additional skill to add to their curriculum vitaes. However, having to ask clients/participants about their HPV infection status/sexual history was considered to be ‘shame job’ (colloquial term for embarrassing). It was suggested that this component of the project might best be covered by a non-Indigenous health member given that Indigenous health practitioners are often related to those they treat; (6) those consulted had vast knowledge of the community and connections with Indigenous persons in all walks of life. Mention was made of the large number of itinerant Indigenous people who came through one consultation site during the summer, ceremonial season, and about how we could work together with them through the temporary housing agencies; (7) feedback was considered to be a critical component of the study; too often research projects or screening initiatives did not do this; (8) the uptake of HPV vaccination among the local Indigenous population had been very low. This was considered in light of the generally low uptake by Indigenous persons of vaccinations for other conditions. When asked if part of our community consultation might involve forums around the role of HPV vaccination in potentially reducing throat cancer risk, the view was that explanation of how HPV infection was connected with the mouth (through oral sex) could be awkward/embarrassing for Indigenous community members. The general consensus was that this aspect would be best covered by non-Indigenous staff.

This initial round of Indigenous community consultations resulted in the development and submission of a funding application to Australia’s leading health and medical research funding agency that reflected the principles of respect and reciprocity. Indigenous community views and advice in both the study design and methodology was included. In the final funding application submission, 50% (nine out of 18) investigators were Indigenous.

Additional Indigenous community consultations were undertaken after funding was awarded, with the six ACCHOs who indicated an interest from the original consultations providing advice on how recruitment might best be undertaken. Suggestions for capacity building of local Indigenous staff were included. The ACCHOs provided letters of support, which were used in the applications for ethical approval (obtained from the University of Adelaide Human Research Ethics Committee and the Aboriginal Health Council of South Australia’s Human Research Ethics Committee). Staff were hired (*n* = 6), with non-Indigenous staff (*n* = 3) undertaking extensive cultural competency training through the Garrudja Aboriginal Consultation company and through study workshops with Indigenous staff. An Indigenous Reference Group (IRG) was established to provide oversight and cultural guidance on recruitment strategies and data collection. This included Indigenous community members, councillors and health workers, and was chaired by an Indigenous health manager. A protocol of the study was subsequently published [9].

### Recruitment approaches

Participants were Aboriginal adults residing in South Australia, who were requested to provide a saliva sample for genetic HPV testing, and to complete a self-reported questionnaire including risk factors for HPV and OPSCC. Eligibility for the study included identifying as Aboriginal and/or Torres Strait Islander, being aged 18 years or above, residing in South Australia and being able to provide signed informed consent. Recruitment was conducted by trained research officers. The ACCHOs were instrumental in recruitment, facilitating both staff to promote the study (and to take part) and rooms in which to collect data. Participants were invited to take part in the study at a location of their choice; in some cases this was in their homes, but most often it was at the ACCHOs. There was extensive travel of study staff around the state throughout the recruitment phase.

### Indigenous capacity building

Indigenous capacity building was a core aim of the project and was facilitated by equal responsibility and constant consultation with the ACCHOs, Indigenous Reference Group, Indigenous research assistants and participants. The Indigenous research assistants, including those who were volunteered by the ACCHOs to help the study team, were trained in research skills, ethics principles, data collection, data checking, data filing and disseminating research findings back to community. There were additionally opportunities for members of ACCHOs to represent the study at national and international meetings.

ACCHOs and study participants were able to bolster their knowledge of HPV infection and OPSCC, and the links between the two, through free and frank conversations with the research team. The knowledge-sharing was two-way, with substantial benefits for the non-Indigenous research staff in being included in Indigenous consultative processes and learning from ACCHOs and study participants.

### Documentation of project staff recruitment experiences and analytical approach

Throughout the community consultation, engagement and recruitment phases, Indigenous and non-Indigenous research staff recorded all face-to-face, telephone, text and email interactions with ACCHOs and, in turn, participants. For each interaction, the date and type, key points, and length of time were recorded. Anecdotal reflections of ACCHO and participant interactions were also recorded. The first (JH) and senior (LJ) authors reviewed the research reflection notes and, using an inductive approach to thematic analysis, identified emergent themes within the data. HPV saliva samples were sent for DNA analysis testing and examined against the self-report questionnaire data.

Residents of the communities in which we recruited normally have oropharyngeal squamous cell carcinoma diagnosed through any dental or medical check-ups they might have. Treatment will be provided through a partnership of ACCHOs and tertiary services typically provided in Adelaide (capital city of South Australia).

## Results

Recruitment occurred between Feb 2018 and Dec 2018, with 1011 participants recruited. The average age was 39.8 years, with 45% aged 40 years or older. Two-thirds (66%) were female and more than 60% resided in non-metropolitan locations. Sixty eight percent had high school or less as their highest educational attainment and just over three quarters (76%) received their income through Centrelink.

There was compelling feedback from the Indigenous community regarding fear of cancer and the need for sensitivity in the language used by the study team. One of the Indigenous research officers said, in regard to community consultation:

*Within our Indigenous communities there is a deep fear of cancer. Our study team have to be sensitive as to how we use language. For example, our study team are aware that some communities would rather use the word ‘throat sickness’ instead of throat cancer. Our study team acknowledge this.*

Many of the participants were wary of research and of researchers in general. This meant the study team needed to be trained specifically in appropriate language to use when engaged in consultation, participant recruitment and data collection. One of the views of a study team member included:

*Our study team needs to engage the participant in language that is understood (plain English). This includes a clear description of what the study is about, how the study evolved, what methods will be used, and to ensure the language used is fully understood by each participant. Ensuring they fully understand and are comfortable about all their rights including privacy and exercising them.*

Feedback from some of the Aboriginal health service staff included having respect for the kind of language used commonly in day-to-day health service interactions: *When I talk about language, it is also important to respect the language we as Indigenous Australians use. A type of English only Indigenous people would relate to.* This has been empirically reported in the peer-reviewed literature [10].

Respect of language was also highlighted by both the study IRG and Indigenous staff: *Our team respect their [*study participants’*] language and how their responses may not suit the traditional research style. Yet, as a researcher and as an Indigenous researcher, it is of the utmost importance to respect the participants’ style of answering or asking questions about the study or the methods used. Participants have the right to ask more questions, make a complaint and understand the preservation of their privacy and confidentiality.*

Being on country is a powerful connector of Aboriginal people from around Australia. This was deeply embedded into the training component of the study’s research officers. Some of the reflections of being ‘on Country’ and community buy-in included:

*With the 1000 participants living in urban, regional or remote areas of South Australia, our researchers travel to the participants’ Country* (In the Indigenous Australian context, connection with ‘Country’ is **of great significance. It goes far beyond physical elements, and is fundamental to identity)***. The study participants allow our research team to be welcomed onto their Country and into their homes or at a local Aboriginal organization. There is a respect for their time. There is a respect for the values each participant has in relation to research. An example of this is the time it takes to complete a questionnaire. Some participants are asking questions. Some give examples, stories of family, friends or of themselves and the health issues that confront them. Telling their story may enable the participant to give a more accurate answer to the questions being asked of them.*

Because experiences of research by many Aboriginal Australians have been far from positive, there is a need for the research relationship to be overtly reciprocal. This was highlighted in some of the feedback from the study team: *There is a sharing or exchange of good will and commitment between our team and the participants. A reciprocal approach to respect of participants’ reasons for participating and why the study is being conducted. A mutual respect and understanding of the future health outcomes of all Indigenous Australians more broadly.*

Our team worked hard to ensure the research partnership was very much 50:50 with research participants. There are some helpful perspectives on why participants wanted to be involved, which include:

*Our study participants have made a choice to be actively involved. To inform the researchers what they feel about health, the health of their Community and family. Most importantly our participants have a fundamental reason for taking part. They have often had family, friends or Community members pass away or been very sick from cancer and in some cases throat sickness. Others just want to be part of creating a new path of wellness for their Community.*

*It’s interesting how participants have embraced a new informed knowledge relating to the Human Papilloma Virus. Some participants have spoken of how they will now go and visit their doctor. A sense of empowerment, being in control to ask more questions of their doctor. Having their doctor undertake investigations, as a preventive measure. Some participants have never undertaken medical investigations before. Others have said that they will let their family members know of this virus and ensure children in their family or Community attend school to have the vaccinations.*

*Others have told how more knowledge needs to be circulated in their local health service. And more information circulated in and among the Indigenous Community for improved understanding of how HPV is linked with throat sickness.*

One positive outcome of participants’ involvement in the research is that many started to see themselves as active agents for change in their community. Perspectives on participants and behavior change include:

*Some participants have seen the study as an opportunity to reflect on their own health in areas of smoking, drinking, diet and exercise, and seeking to improve their current behaviours in relation to this. That is self-determination right there.*

Themes of the engagement, consultation and recruitment processes that appeared to resonate most with community were conceptualised into five domains: (1) engaging with ACCHOs as equal partners very early in the research process; (2) having an Indigenous Reference Group; (3) ACCHOs actively promoting the study; (4) having a flexible agenda responsive to broader environment demands and; (5) including Indigenous capacity building as part of the project.

### Engaging with ACCHOs as equal partners early in research process

Engaging with ACCHOs to work in partnership to formulate research questions and study design 2 years before grant submission was seen as being critical in ensuring the eventual funding application was grounded in an authentic Indigenous voice. In turn, it helped to establish credibility of the study and study team in the community once the recruitment phase commenced.

### Having an indigenous reference group

The Indigenous Reference Group comprised those experienced in community engagement, health and research. It facilitated the research team by providing effective strategic advice and by promoting the study to their wider networks. The Indigenous Reference Group had a key role in shaping the study and providing ongoing guidance including issues relating to cultural sensitivity.

### ACCHOs actively promoting the study

The ACCHOs were the powerhouse behind our large recruitment numbers in a short recruitment period. Because of the extensive community consultation that had occurred prior to grant submission, there was a sense of partnership with the study and substantial buy-in and support. There was a genuine belief in the importance of the research questions, the study design, and the potential benefits the study might have in shaping future policies around HPV infection and OPSCC among Indigenous Australians.

### Having flexible agenda responsive to broader environment demands

There were many competing demands at a community level at any given time during our recruitment phase. This included, but was not limited to, cultural events, sorry business (deaths, funerals, grieving), inclement weather, high demand for ACCHO services, and limitations on ACCHO staff availability to help. We were able to align our travel times and recruitment strategies to mitigate against this.

### Including indigenous capacity building as part of project

ACCHO staff were highly receptive to learning about the various study-related activities, including the informed consent processes and dissemination of study findings. This was seen as something that could be added to the curriculum vitaes of the volunteers who helped, thus furthering their own potential research careers. In addition, it was advantageous to the ACCHOs in terms of having an increased understanding of research processes. This was seen to be helpful when approached by external groups to be involved in future research initiatives.

With respect to the CONSIDER guidelines (Table 1), key areas of strength were documented against each of the eight domains. These included: formal letters of support from participating ACCHOs (governance); extensive community engagement and consultation to refine the study aims (prioritization); community consultation repeated once funding was received, with ACCHOs providing structures, strategies and recommendations (relationships); all data de-identified, with field staff not analysing data once entered in database (methodology); participants aware, during the informed consent process, of the time commitments involved in being part of the study (participation); Indigenous research assistants, including those volunteered by ACCHOs, trained in research skills, ethics principles, data collection, data checking, data filing and disseminating research findings back to community (capacity); key Indigenous stakeholders and Indigenous researchers contributing as co-authors in publications, enabling Indigenous values and perspectives to contribute to interpretation of the study findings (analysis and findings); and presentations at international conferences by the Indigenous project manager and two ACCHO staff (dissemination).

## Discussion

The findings portray the engagement, consultation and recruitment strategies used in a study involving oral HPV infection and OPSCC among Indigenous South Australians. Five domains were identified and included engaging with ACCHOs early in the research process, having an Indigenous Reference Group, having ACCHOs actively promoting the study, having a flexible agenda and including Indigenous capacity building. From the outset, the project was governed and directed by the Indigenous Australian community, through formation of the Indigenous Reference Group. The project embraced novel suggestions of the ACCHOs, participants, Indigenous research assistants and the Indigenous Reference Group in regards to ways of communicating study-related information, using humour and keeping messages context-specific. All stages of the project were discussed with the ACCHOs, with suggestions from members being considered with equal weighting as those from the study investigators. Strengths in each of the CONSIDER statement’s criteria were identified.

In their systematic review of strategies for improving health research outcomes among socially vulnerable populations, Bonevski and colleagues [11] concluded that researchers needed to build in extended timeframes, have adjustable recruitment protocols, plan for higher resourcing costs and operate via community partnerships. However, administrators of funding grants and research institutions usually operate within tight fiscal parameters, meaning extended timelines and the costs associated with this (particularly for community engagement), need to be factored into the initial grant application. But resources need to be available for community consultation far in advance of any proposal being submitted; resources that, in an increasingly competitive grant funding environment, may be difficult to appropriate. Not having the resources or time to adequately consult with community ultimately means the outcomes are compromised and possibly not reflective of a true partnership (or of what the Indigenous communities were seeking in terms of health benefits/knowledge). It is important that any partnerships with Indigenous communities in a health research capacity is fully cognisant of this.

The Australian Human Rights Commission defines self determination of Indigenous Australians as an ongoing process of choice to ensure Indigenous communities are able to meet their social, cultural and economic needs [12]. Part of this self-determination is the choice to be involved, in truly bipartisan partnerships, in health-related research that is driven by community aspirations and recognised need. In Australia, this is certainly facilitated by the country’s largest health funding organisation (the National Health and Medical Research Council), who commit at least 5 % of its medical research endowment account to Indigenous health [13]. There are clear expectations that such research meets the criteria of community engagement, benefit, sustainability and transferability, and building capability. Such applications are reviewed by an Indigenous Grant Review Panel, which comprises Indigenous leaders in health research from across the country.

The CONSIDER statement provided a useful framework against which to ensure the study team met recommended guidelines for strengthening the reporting of research that explicitly involves Indigenous persons. As stated by the framework’s developers, strengthening research responsiveness is essential in addressing health equity [4], which is especially relevant when addressing health inequities between Indigenous and non-Indigenous Australians. The use of such a tool helps increase research accountability, with adherence to the criteria ultimately strengthening the research process that will hopefully lead to positive and productive impacts on future health policy and translational outcomes. Specific lessons learned from this study that may be of value for other communities, and indeed potentially make the results generalizable, include the need for active and wide community consultation that is initiated very early in the research process, strong and sustained capacity building and an active and engaged Indigenous Reference Group. Results of this study can be disseminated through feedback to the Indigenous communities involved, presentations by the Indigenous research officers at both national and international conferences and involvement in all forms of media.

## Conclusions

In conclusion, consultation and engagement with all sectors of the Indigenous community are essential early in the research phase. Enabling local Indigenous staff to provide cultural guidance throughout the research process is crucial. Our findings suggest that research that is culturally respectful, and delivered in partnership with Indigenous groups, can be embraced when the research is collaborative and has clear translational benefits. The CONSIDER statement is a useful checklist against which to assess Indigenous health research processes, making them more transparent, translatable and accountable. In future, the findings may be useful to yield important Aboriginal population estimates for both oral HPV infection and OPSCC. This may serve to convince funding bodies to provide health promotion personnel in the field of oral health, specifically OPSCC, in ACCHOs.

## Data Availability

Available from corresponding author upon request.

## Abbreviations

ACCHO: Aboriginal Community Controlled Health Organisation
CONSIDER statement: Consolidated Criteria for Strengthening Reporting of Health Research involving Indigenous Peoples
HPV: Human Papillomavirus
IRG: Indigenous Reference Group
NHMRC: National Health and Medical Research Council
OPSCC: Oropharyngeal Squamous Cell Carcinoma
